# The relationship between hippocampal subfield volumes and autobiographical memory persistence

**DOI:** 10.1002/hipo.23293

**Published:** 2020-12-15

**Authors:** Daniel N. Barry, Ian A. Clark, Eleanor A. Maguire

**Affiliations:** ^1^ Wellcome Centre for Human Neuroimaging, UCL Queen Square Institute of Neurology University College London London UK

**Keywords:** autobiographical memory, episodic, hippocampus, longitudinal, pre/parasubiculum, subfields, volume

## Abstract

Structural integrity of the human hippocampus is widely acknowledged to be necessary for the successful encoding and retrieval of autobiographical memories. However, evidence for an association between hippocampal volume and the ability to recall such memories in healthy individuals is mixed. Here we examined this issue further by combining two approaches. First, we focused on the anatomically distinct subregions of the hippocampus where more nuanced associations may be expressed compared to considering the whole hippocampal volume. A manual segmentation protocol of hippocampal subregions allowed us to separately calculate the volumes of the dentate gyrus/CA4, CA3/2, CA1, subiculum, pre/parasubiculum and uncus. Second, a critical feature of autobiographical memories is that they can span long time periods, and so we sought to consider how memory details persist over time by conducting a longitudinal study whereby participants had to recall the same autobiographical memories on two visits spaced 8 months apart. Overall, we found that there was no difference in the total number of internal (episodic) details produced at Visits 1 and 2. However, further probing of detail subcategories revealed that specifically the amount of subjective thoughts and emotions included during recall had declined significantly by the second visit. We also observed a strong correlation between left pre/parasubiculum volume and the amount of autobiographical memory internal details produced over time. This positive relationship was evident for particular facets of the memories, with remembered events, perceptual observations and thoughts and emotions benefitting from greater volume of the left pre/parasubiculum. These preliminary findings expand upon existing functional neuroimaging evidence by highlighting a potential link between left pre/parasubiculum volume and autobiographical memory. A larger pre/parasubiculum appears not only to protect against memory decay, but may possibly enhance memory persistence, inviting further scrutiny of the role of this brain region in remote autobiographical memory retrieval.

## 1 INTRODUCTION

Structural integrity of the hippocampus has long been associated with the formation and retrieval of episodic memories. Neurosurgical resection (Scoville & Milner, 1957), as well as encephalitic (Miller et al., 2017, 2020), neurodegenerative (Petersen et al., 2000; Zhao et al., 2019), epileptic (Reminger et al., 2004), and psychiatric (Herold et al., 2013; Vythilingam et al., 2004) pathologies lead to reductions in hippocampal volume with concomitant memory impairments. However, the evidence for an association between hippocampal volume and episodic memory in healthy individuals is mixed (Van Petten, 2004; see Clark et al., 2020 for a recent full discussion). Some of these inconsistencies may be driven by the complex developmental trajectory of hippocampal structure (Tamnes, Bos, van de Kamp, Peters, & Crone, 2018), and ageing‐related atrophy (Gorbach et al., 2017; Langnes et al., 2020; Nordin, Herlitz, Larsson, & Söderlund, 2017). However, even when age is taken into account, factors remain which may have obscured relationships between hippocampal volume and memory in previous investigations of healthy people.

For instance, while links between the volume of the whole hippocampus and performance on memory tasks have been difficult to establish consistently, the hippocampus comprises anatomically distinct subregions where more nuanced associations may be expressed. Indeed, some effects have been reported. These include, for example, larger CA3 volume associated with reduced subjective confusion when recalling highly similar memories of movie stimuli (Chadwick, Bonnici, & Maguire, 2014; see also Doxey & Kirwan, 2015), and verbal memory ability being positively associated with CA1/subiculum surface area, as measured by the number of hippocampal dentations (Fleming Beattie et al., 2017). Therefore, a focus on hippocampal subregions may be one potentially promising avenue to pursue, and their accurate delineation on structural MRI scans is best achieved using manual segmentation (Dalton, Zeidman, Barry, Williams, & Maguire, 2017; Olsen et al., 2019; Yushkevich et al., 2015).

Another factor that may contribute to the relatively weak associations between hippocampal volume and memory task performance in healthy people is a lack of ecological validity. Studies often use simple, controlled laboratory‐based memory tasks, such as recalling lists of words (Van Petten, 2004). By contrast, in real‐world contexts, the hippocampus is involved in the retrieval of multi‐faceted, multi‐modal autobiographical memories of our personal past experiences (Steinvorth, Levine, & Corkin, 2005). However, even when autobiographical memory recall was examined in a wide‐ranging analysis of healthy people, Clark, Monk, Hotchin, et al. (2020) did not find any significant relationship between overall hippocampal volume and recall of internal (episodic) details, a widely‐used measure from the Autobiographical Interview (Levine, Svoboda, Hay, Winocur, & Moscovitch, 2002). To the best of our knowledge, only one study has examined the links between hippocampal subregion volumes and autobiographical memory recall in healthy individuals, reporting a positive association between the number of internal details on the Autobiographical Interview (Levine et al., 2002) and the volume of both the left subiculum and the combined left dentate gyrus (DG)/CA2/CA3 region (Palombo et al., 2018). The authors of that paper speculated that, given the reported relationship between the volume of CA3 and the ability to recall episodic memories with precision (Chadwick et al., 2014) and ease (Hebscher, Levine, & Gilboa, 2018) in healthy people, and in rich detail in patients (Miller et al., 2017), the volume of this region alone may be driving the latter association.

Aside from ecological validity, a critical feature of real‐world autobiographical memories is that they can span decades, and simpler laboratory‐based measures of memory often fail to capture this longevity of mnemonic representations. Nevertheless, even among laboratory‐based tasks, such as recalling word lists, there is accumulating evidence that, when it concerns volume, memory persistence may be important to consider. Stronger associations between hippocampal volume and memory retrieval ability seem to emerge when there is a delay between learning and retrieval, whether this is on the order of minutes (Pohlack et al., 2014), half an hour (Poppenk & Moscovitch, 2011), 1 week (Ostby, Tamnes, Fjell, & Walhovd, 2012), 10 days (Fjell et al., 2019), or 11 weeks (Walhovd et al., 2004). Hence, there may be much to learn from longitudinal studies, but they are often more challenging to perform than cross‐sectional experiments.

We sought to build on the findings of Palombo et al. (2018) by investigating the relationship between the degree of long‐term persistence of autobiographical memories and the volume of specific hippocampal subregions. To address this issue, we conducted a longitudinal study whereby we asked participants to recall the same autobiographical memories on two separate visits spaced 8 months apart. We used the Autobiographical Interview protocol (Levine et al., 2002) to score the memories and combined this with a comprehensive manual segmentation protocol of hippocampal subregions (Dalton et al., 2017). This allowed us to investigate the relationship between the separate volumes of the DG/CA4, CA3/2, CA1, subiculum, pre/parasubiculum and uncus, and the autobiographical memory details produced over an extended time period.

We had two hypotheses. First, given Palombo et al.'s (2018) finding of an association between the volume of the combined DG/CA2/CA3 region and the recall of internal details, the role of the DG in disambiguating representations in memory (Berron et al., 2016), and the contribution of CA3 to completing holistic representations (Grande et al., 2019), we predicted that DG and/or CA3 volume may be related to the preservation of event details over an extended period of time. The manual segmentation protocol deployed here enabled us to separate these two regions and determine their individual contributions to the persistence of autobiographical memory.

Our second hypothesis related to subregions of the hippocampus that are often not segmented in isolation in volumetric studies, the presubiculum and parasubiculum. Typically, these areas are subsumed within a broader subiculum mask (e.g., Palombo et al., 2018), even though they can be differentiated from the subiculum by specific structural characteristics that can be visualized on either histological slices or high‐resolution (i.e., sub‐millimeter voxel size) MRI scans (Dalton & Maguire, 2017; Ding & Van Hoesen, 2015; Green & Mesulam, 1988). The boundary between the presubiculum and parasubiculum, however, cannot be reliably delineated on 3T MRI scans and so here the two are combined into one subregion, the pre/parasubiculum. Specific activation of the pre/parasubiculum has been observed during functional MRI (fMRI) studies of autobiographical memory retrieval (e.g., Addis, Knapp, Roberts, & Schacter, 2012; see also Zeidman, Lutti, & Maguire, 2015; Dalton, Zeidman, McCormick, & Maguire, 2018; and the review of Zeidman & Maguire, 2016). It has also been noted that the pre/parasubiculum has privileged access to holistic representations of the environment, which are central to autobiographical memories, and it may therefore be neuroanatomically pre‐disposed to be involved in processing such memories (Dalton & Maguire, 2017). Consequently, we hypothesized that the amount of autobiographical memory internal details produced after a considerable delay might be related to the volume of the pre/parasubiculum, and that the positive association found between the subiculum volume and autobiographical memory reported by Palombo et al. (2018) may have been driven by the pre/parasubiculum.

## 2 METHODS

### 2.1 Participants

Sixteen right‐handed participants (14 female, mean age 24.7 years, *SD* 3.1, range 21–33) took part in the experiment. All had normal or corrected‐to‐normal vision. The study was approved by the University College London Research Ethics Committee (approval reference 6743/002). Written informed consent was obtained from each participant. The overall autobiographical memory internal and external details scores have been reported before in a study focused on a different research question (Barry, Chadwick, & Maguire, 2018). The internal details subcategories data and the structural MRI data have not been published previously.

### 2.2 Selection of autobiographical memories and memory interviews

#### 2.2.1 Visit 1

**FIGURE 1:**
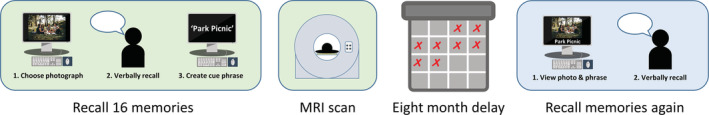
Longitudinal experimental design. During a participant's initial visit, they recalled 16 memories with the aid of personal photographs, and for each memory chose a cue phrase to aid in subsequent recall. One week later they underwent a structural MRI scan. Following an 8‐month delay, they returned to recall the same memories again, using the photographs and previously‐selected cue phrases to assist recall [Color figure can be viewed at wileyonlinelibrary.com]

To assist in the selection of specific autobiographical memories and to ensure memory age was controlled across our sample, participants were instructed to choose from their own collections at least three digital photographs corresponding to each of eight time points in their past (2 weeks, 4 months, 8 months, 12 months, 16 months, 20 months, 24 months and 5 years) relative to the time of taking part in the experiment. These photographs served to remind participants of vivid, unique, and specific autobiographical events (Figure 1 left). Photographs were chosen from the participants' pre‐existing photograph collections and not prospectively taken with the study in mind. Highly personal, emotionally negative, or repetitive events were deemed unsuitable. An additional requirement was that memories from the same time period should be dissimilar in content.

During autobiographical memory recall, which was recorded and subsequently transcribed, participants were asked to describe in as much detail as possible the specific autobiographical memory elicited by their chosen photograph. General probes were given by the interviewer when appropriate (e.g., “what else can you remember about this event?”). Participants rated each memory on a number of characteristics, and two memories from each time period (16 memories in total) where participants indicated high vividness, detail and ease of recall were selected for inclusion in the experiment. Specifically, on a vividness scale where one was “not at all vivid” and five was “highly vivid,” the mean rating across all memories was 4.13 (*SD* 0.35). On a scale of detail where one was “not at all detailed” and five was “highly detailed,” the mean rating was 3.86 (*SD* 0.47). On a scale of ease of recall, where one was “not at all difficult” and five was “highly difficult,” the selected memories had a mean rating of 1.68 (*SD* 0.31). For emotional valence, a rating of 1–2 was negative, 3 neutral, 4–5 positive; the mean was 4.45 (*SD* 0.27). Participants created a short phrase pertaining to each memory which was paired with the photograph to facilitate recall during the subsequent fMRI experiment reported in Barry et al. (2018).

#### 2.2.2 Visit 2

Participants had been informed during Visit 1 that they may be contacted about attending for a second visit, but they were naïve about the aims and demands of the follow‐up experiment. The 16 participants returned for Visit 2 approximately 8 months later (mean 8.4 months, *SD* 1.2). They were presented with their 16 photographs and cue phrases associated with the autobiographical memories from Visit 1 and were asked to describe in as much detail as possible the specific events that they had recalled previously (Figure 1 right), and to perform the same ratings as during Visit 1 (such as vividness). General probes were used by the experimenter where appropriate (e.g., “what else can you remember about this event?”). The interviewer availed of summarized transcripts from Visit 1 to verify the same memory and details were being recalled. The memory interview during this second visit was also recorded and transcribed. To ensure consistency in the delivery of the Autobiographical Interview, it was conducted by the same experimenter during both visits.

### 2.3 Behavioral analyses

The autobiographical memory interviews were recorded and transcribed to facilitate an objective analysis of the details using the Autobiographical Interview protocol (Levine et al., 2002). Details provided for each memory were scored as either “internal” (episodic) or “external” (semantic). Internal details were composed of five subcategories: event details referred to happenings, specific individuals present, weather conditions, actions which were physical or emotional, or reactions elicited in others. Time details referred to the time of day, week, month, season or year. Place details were composed of references to an event location, such as room or part within, building, street or city. Perceptual details concerned auditory, olfactory, tactile, taste, and visual features, as well as body position and duration. Thoughts which occurred to the participant during the original experience, as well as subjective emotional states and their implications were coded as thoughts and emotions. External details consisted of any references to details from events other than the one being recalled, general knowledge or facts, events which were ongoing rather than specific to a particular time, or an extended state of being. Details that were repeated without solicitation and metacognitive statements, or editorializing, were also coded as external details.

One rater performed the scoring across both time‐points to ensure consistency. In addition, a subset of 16 memories (*n* = 2 per time period) were randomly selected across the 16 participants and scored by another experimenter blind to the aims and conditions of the study. Intraclass coefficient estimates were calculated using SPSS statistical package version 22 (SPSS, Chicago, IL) based on a single measure, absolute‐agreement, two‐way random‐effects model. Inter‐rater reliabilities for the scoring were high for both internal (intraclass correlation [ICC] = 0.94) and external (ICC = 0.81) details.

To generate a robust, global measure of episodic memory recall for each participant, we summed the recalled internal details across all 16 memories within each visit. Differences in total internal details produced at Visit 1 and Visit 2 were analyzed using a paired *t* test. Further analyses of the differences within each internal detail subcategory across time were also assessed using paired *t* tests, with an adjusted *p* value threshold of *p* < .01 to correct for multiple comparisons. In addition, we calculated the change in the number of memory details produced over time for each participant. This was the difference in the number of memory details from Visit 1 to Visit 2 expressed as a ratio measurement, where a score of one meant the participant produced the exact same number of details during both visits, a score above one indicated the participant produced more memory details during the second visit, and a score below one represented a loss of details over time. This was calculated for the overall number of internal details, as well as each subcategory of internal details. This ratio measurement over time not only provided a single score which could be associated with hippocampal subregion volumes, but also served to control for participant verbosity across the group. Moreover, this measure was insensitive to whether some participants tended to produce more details in general, and quantified the key variable of interest which was the *within‐participant* change over time.

As participants were instructed to recall specific and unique autobiographical events, as opposed to unrelated events or facts, external details were not considered a suitable measure of verbosity, and were more likely an index of task compliance in this study. In addition, these details did not provide any insight into the persistence of specific autobiographical memory details over time, and therefore were not incorporated into the current analysis. However, for completeness, and to demonstrate equivalent compliance to task demands across the two visits, a paired samples *t* test did not reveal any statistically significant difference (*t*_15_ = −0.5, *p* = .622) between the mean total number of external details produced at Visit 1 (107.19, *SD* 38.06) and Visit 2 (111.56, *SD* 54.72).

Participants were also asked how frequently they had thought about the autobiographical memories before Visit 1, and between the two visits. Their responses were recorded on a scale from one (never) to five (very frequently). The mean response at Visit 1 was 2.80 (*SD* 0.40), indicating that they had not thought about the memories that much since the original events had occurred. Interestingly, when at Visit 2 they were asked how much they had thought about the memories during the 8 months between visits, this rating had dropped even further to 1.78 (*SD* 0.52), a change which was significant (*t*_15_ = 8.08, *p* < .001).

As described above, during both visits participants were asked to rate each memory in terms of its level of vividness, detail, ease of recall, and emotional valence. Ratings were also obtained of their personal significance on a scale of one (low) to five (high), and the perspective from which memories were recalled, whether first (1) or third person (2). After 8 months had elapsed, participants rated their memories as less vivid (4.13, *SD*: 0.35 vs. 3.27, *SD*: 0.48; *t*_15_ = 9.41, *p* < .001), less detailed (3.86, *SD*: 0.47 vs. 3.04, *SD*: 0.39; *t*_15_ = 11.26, *p* < .001), less easy to recall (1.68, *SD*: 0.31 vs. 2.25, *SD*: 0.49; *t*_15_ = −6.59, *p* < .001), less positive (4.45, *SD*: 0.27 vs. 4.19, *SD*: 0.35; *t*_15_ = 3.13, *p* = .007), and less personally significant (3.34, *SD*: 0.48 vs. 2.94, *SD*: 0.55; *t*_15_ = 3.44, *p* = .004). Memories did not differ significantly in terms of the perspective from which they were recalled (1.08, *SD*: 0.12 vs. 1.1, *SD*: 0.16; *t*_15_ = −0.72, *p* = .485). None of these subjective ratings correlated significantly with any of the hippocampal subregion volumes.

### 2.4 MRI data acquisition

One week following their first visit during which the autobiographical memories were selected and recalled, participants were scanned using a structural MRI sequence which was optimized for high‐resolution imaging of the hippocampus. Images were acquired using a 3 Tesla MRI system (Magnetom TIM Trio, Siemens Healthcare, Erlangen, Germany), within a partial volume that incorporated the entire extent of the hippocampal formation. Data were collected using a single‐slab 3D T2‐weighted turbo spin echo sequence with variable flip angles (SPACE) (Mugler et al., 2000) in combination with parallel imaging to simultaneously achieve a high image resolution of ~500 μm, high sampling efficiency, and short scan time while maintaining a sufficient signal‐to‐noise ratio (SNR). After excitation of a single axial slab, the image was read out with the following parameters: resolution = 0.52 × 0.52 × 0.5 mm, matrix = 384 × 328, partitions = 104, partition thickness = 0.5 mm, partition oversampling = 15.4%, field of view = 200 × 171 mm, echo time (TE) = 353 ms, TR = 3,200 ms, GRAPPA × 2 in phase‐encoding (PE) direction, bandwidth = 434 Hz/pixel, echo spacing = 4.98 ms, turbo factor in PE direction = 177, echo train duration = 881, averages = 1.9. For reduction of signal bias due to, for example, spatial variation in coil sensitivity profiles, the images were normalized using a prescan, and a weak intensity filter was applied as implemented by the scanner's manufacturer. To improve the SNR of the anatomical image, three scans were acquired for each participant, which were coregistered, denoised and averaged.

### 2.5 Segmentation of hippocampal subfields

**FIGURE 2:**
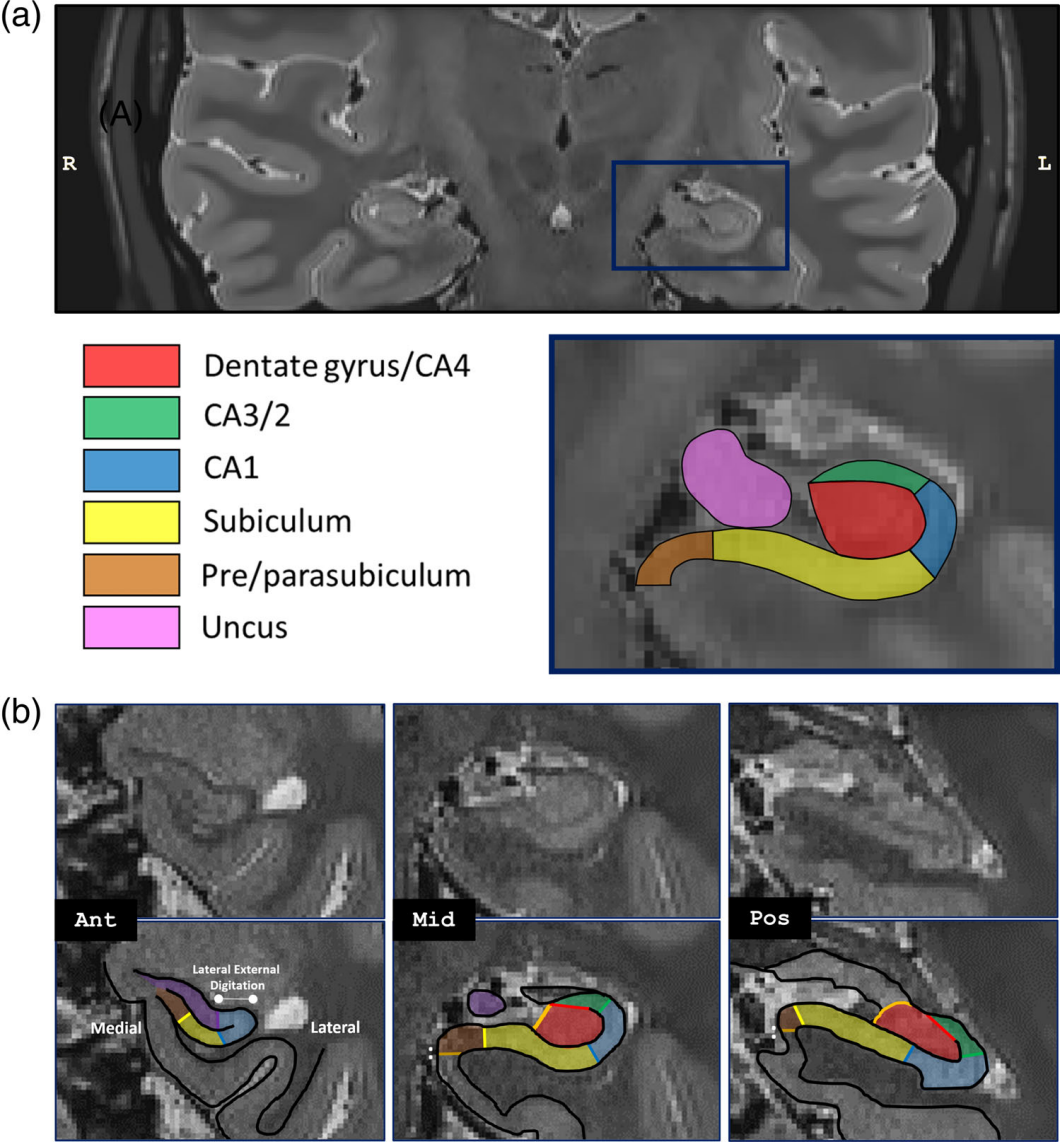
Example hippocampal segmentation of a participant. (a) A representative high resolution (0.5 mm^3^) T2‐weighted coronal slice (upper panel) is displayed in native space, with a segmentation (lower panel) of the left hippocampus into its subregions, based on the protocol of Dalton et al. (2017). The displayed coronal slice is located toward the posterior end of the anterior hippocampus. Note that in native space, the right side is shown on the left. (b) The segmentation protocol is shown for the anterior, middle and posterior hippocampus without and with subregion delineations overlaid (adapted from Dalton et al., 2017). Focusing specifically on the pre/parasubiculum, this region emerges anteriorly where the lateral portion of the hippocampus bends dorsally (Ant). The lateral boundary of the pre/parasubiculum with the subiculum can be identified as a region of relatively darker gray matter on T2‐weighted images, due to dense innervations from the perforant pathway. Toward the posterior hippocampus (Mid‐Pos), there is a gradual lateral to medial shift in the location of this border. The medial border of the anterior pre/parasubiculum is located at the ventromedial edge of the hippocampus (Ant). From the appearance of the uncul sulcus onwards (Mid‐Pos), which splits the hippocampus into dorsal and ventral components, the medial border of the pre/parasubiculum occurs at the location where the medial extent of the subicular cortices turns sharply in a ventral direction (see “:”) [Color figure can be viewed at wileyonlinelibrary.com]

We manually delineated left and right hippocampal subfields on participants' structural MR images in native space according to the methodology outlined by Dalton et al. (2017), using ITK Snap software version 3.6.0 (Yushkevich et al., 2006). The following subfields were segmented: DG/CA4, CA3/2, CA1, subiculum, pre/parasubiclulum and uncus (Figure 2). To assess inter‐rater reliability, a second experimenter independently segmented the left and right hippocampi of four of the participants (25% of the data). Reliability of segmentations was assessed using the DICE metric (Dice, 1945) to produce a score between 0 (no overlap) and 1 (perfect overlap). Inter‐rater reliability for the left hippocampus was 0.86 for DG/CA4, 0.70 for CA3/2, 0.75 for CA1, 0.78 for subiculum, 0.68 for pre/parasubiculum, and 0.81 for the uncus. In the right hippocampus, the inter‐rater reliabilities were as follows: 0.85 for DG/CA4, 0.64 for CA3/2, 0.72 for CA1, 0.77 for subiculum, 0.63 for pre/parasubiculum, and 0.78 for the uncus. These values are equivalent to those reported in the extant literature (e.g., Bonnici, Chadwick, & Maguire, 2013; Chadwick et al., 2014; Dalton, McCormick, De Luca, Clark, & Maguire, 2019; Palombo et al., 2013).

### 2.6 Correlations between hippocampal subregion volumes and amount of memory details

Table 1 displays the volumes of the left and right hippocampal subregions. To assess if there was a relationship between hippocampal subregion volumes and individual differences in autobiographical memory details across time, we performed partial correlations between the subregion volumes and the ratio of internal details produced from Visit 1 to Visit 2, with age, gender and total hippocampal volume as covariates. This involved each of the six subregions in each hemisphere, and the *p* value threshold for significance was adjusted accordingly to 0.004. To examine the association between memory details and the size of the left pre/parasubiculum (the main result from the previous correlation analysis), we performed additional partial correlation analyses involving the five subcategories of internal memory details, with age, gender and overall hippocampal volume as covariates, with the *p* value threshold adjusted to 0.01 to account for multiple comparisons.

**TABLE 1:** Left and right hippocampal subregion volumes in mm^3^ (mean, *SD*)

	Left	Right
Dentate gyrus/CA4	589.46 (96.49)	543.96 (51.59)
CA3/2	144.33 (30.86)	138.36 (24.53)
CA1	565.69 (88.56)	590.18 (92.79)
Subiculum	650.89 (102.83)	649.23 (87.69)
Pre/parasubiculum	320.99 (46.18)	241.79 (52.92)
Uncus	501 (89.07)	588.36 (147.18)

## 3 RESULTS

### 3.1 Changes in memory details after 8 months

Although participants' self‐reported ratings indicated that, from a subjective perspective, they regarded their memories as less detailed overall after 8 months, objective analyses revealed no statistically significant changes in the total number of internal details produced between Visit 1 and Visit 2 across the group (Table 2). However, analyses of the five subcategories of internal details showed that participants produced significantly fewer details about subjective thoughts and emotional states during Visit 2 compared to 8 months previously (*t*_15_ = 3.21, *p* = .006).

**TABLE 2:** Total internal details (mean, *SD*) for all autobiographical memories

	Visit 1	Visit 2 (+8M)	*t*	*p*
All internal details	266.75 (61.46)	291.19 (87.67)	−1.62	.126
Event	156.94 (39.47)	180 (58.28)	−2.4	.030
Time	9.56 (3.90)	13.69 (6.12)	−2.61	.020
Place	28.13 (7.94)	31.88 (11.47)	−1.83	.087
Perceptual	39.63 (13.70)	40.88 (19.48)	−0.37	.719
Thought/emotion	32.50 (11.49)	24.75 (12.60)	3.21	.006*

^*^ Indicates a statistically significant difference between visits at a *p* value threshold of .01 (adjusted for multiple comparisons across subcategories of internal details).

**FIGURE 3:**
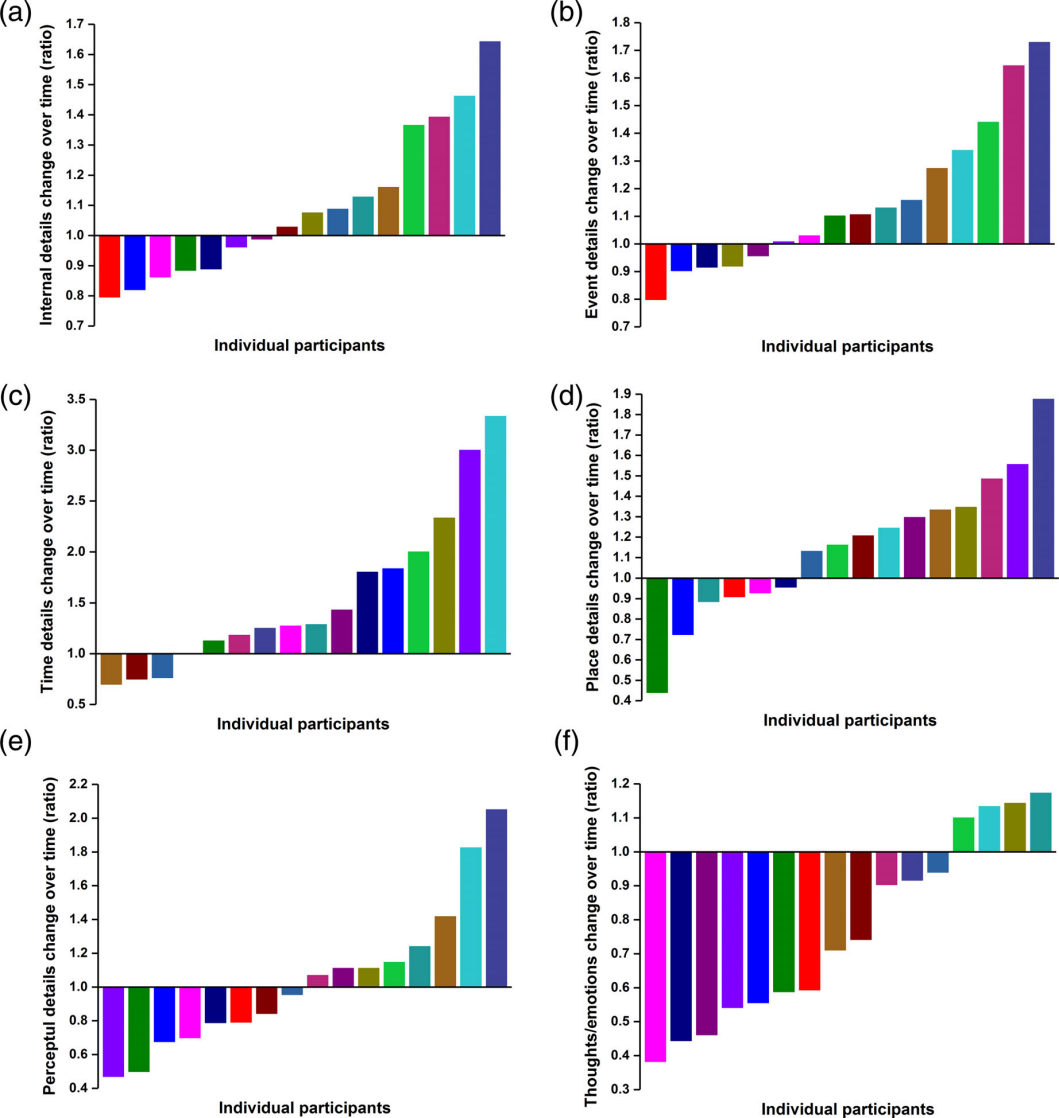
Time‐dependent changes in the amount of autobiographical memory details produced after an 8‐month delay for each participant. Each participant is consistently represented by a unique color across all graphs. A ratio of one indicates a participant produced the same amount of details during both visits, a ratio above one indicates more detail was produced during the second visit, while a ratio below one indicates a decline in the amount of details over the 8‐month period. Ratios for each participant are ordered from lowest to highest in each graph to facilitate a clear interpretation of the distribution of scores across the group. Panel (a) represents total internal details, while panels (b–f) display the memory recall changes within each subcategory of internal details [Color figure can be viewed at wileyonlinelibrary.com]

We then quantified the change in internal details produced across time for each individual participant, by expressing it as a ratio of generated details from Visit 1 to Visit 2. Figure 3 displays the individual ratio scores for each participant for the total internal details and then separately for each subcategory of internal details. This demonstrated that while the group, on average, did not differ in terms of the number of internal details produced between the two visits, there was considerable variation across the participants. Approximately two thirds of the participants produced more internal details following an 8‐month delay (Figure 3a). This pattern was also evident for specific event details (Figure 3b), as well as references to time (Figure 3c) and place (Figure 3d). An equivalent number of participants displayed loss of perceptual details as those who produced more details over time (Figure 3e), while subjective thoughts and emotions were consistently vulnerable to decay over time, with the majority of participants generating fewer of these details during their second visit (Figure 3f).

### 3.2 Correlations between hippocampal subregion volumes and memory details across time

**FIGURE 4:**
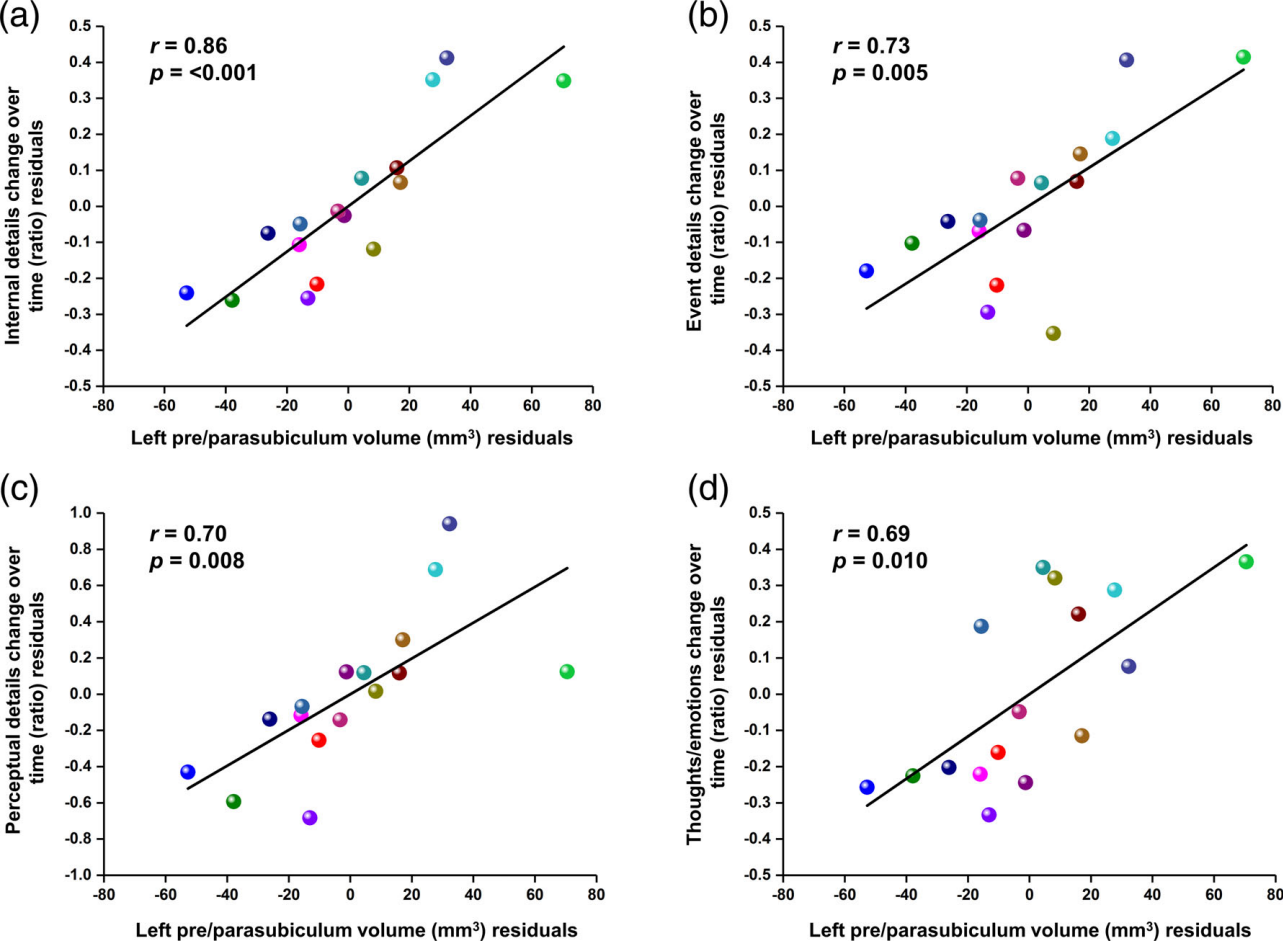
Partial correlation plots showing the association between the left pre/parasubiculum volume and time‐dependent changes in the amount of autobiographical memory details produced. Total internal details are plotted in (a), followed by event details (b), perceptual details (c) and thoughts and emotions (d). The plotted values represent the correlation between the residuals of the change in memory details over time and the residuals of the pre/parasubiculum volume after controlling for age, gender and total hippocampal volume, and are centered around zero. Each participant is represented by the same color as that displayed in Figure 3 [Color figure can be viewed at wileyonlinelibrary.com]

The core research question of this study was whether an association was present between hippocampal subregion volumes and autobiographical memory details across a considerable delay. A significant correlation between volume and amount of internal details was found in only one subregion, the left pre/parasubiculum; this was strong and positive (*r* = .86, *p* < .001; Table 3; Figure 4a). We also observed a moderate positive correlation between right CA3/2 volume and internal details over time, but this did not survive correction for multiple comparisons.

**TABLE 3:** Partial correlations between the total internal details change across time and hippocampal subregion volumes, with age, gender and total hippocampal volume as covariates

	Left (*r*)	*p*	Right (*r*)	*p*
Dentate gyrus/CA4	−.19	.537	.28	.357
CA3/2	−.23	.453	.73	.005
CA1	.25	.405	.09	.765
Subiculum	.57	.041	−.55	.051
Pre/parasubiculum	.86	<.001*	.06	.846
Uncus	.37	.218	.16	.594

^*^ Indicates statistically significant correlations between hippocampal subregion volumes and memory recall over time at a *p* value threshold of .004 (adjusted for the number of subregions across both hemispheres).

To examine the association between memory details and the volume of the left pre/parasubiculum further, we performed additional correlational analyses on the subcategories of internal memory details (Table 4). We found a significant positive correlation with specific event details over time (*r* = .73, *p* = .005; Figure 4b), perceptual observations (*r* = .7, *p* = .008; Figure 4c), and thoughts and emotions (*r* = .69, *p* = .01; Figure 4d). While we did not find a significant relationship with time details, we noted that the correlation between left pre/parasubiculum volume and references to places was close to the threshold for significance (Table 4).

**TABLE 4:** Partial correlations between the change in recall of internal details subcategories across time and volume of the left pre/parasubiculum, with age, gender and total hippocampal volume as covariates

	*r*	*p*
Event	.73	.005*
Time	.15	.619
Place	.66	.014
Perceptual	.70	.008*
Thought/emotion	.69	.010*

^*^ Indicates statistically significant correlations between pre/parasubiculum volume and memory recall over time at a *p* value threshold of .01 (adjusted for the number of internal details subcategories).

## 4 DISCUSSION

This longitudinal study examined the relationship between individual differences in the amount of details produced when recalling the same autobiographical memories over a considerable delay and hippocampal subfield volumes. On average across the group, there was no difference in the total number of internal (episodic) details produced at Visits 1 and 2 which were 8 months apart. However, further probing of detail subcategories revealed that specifically the amount of subjective thoughts and emotions that were reported during the first visit declined significantly over time. Examination of each individual's change in performance between visits, identified a strong correlation between left pre/parasubiculum volume and the ratio of autobiographical memory internal details over time. This positive relationship was observed for particular facets of the memories, with remembered events, perceptual observations and thoughts and emotions benefitting from greater volume of the left pre/parasubiculum. We consider the behavioral and neuroimaging findings in turn.

Given that people often recall fewer autobiographical memories from remote periods (Rubin, 1982), our finding of preserved detail for specific memories over time appears surprising, but is not without precedence in the literature. In fact, an increase in generated details for the same autobiographical memories has been observed at an even longer delay of one and a half years (Campbell, Nadel, Duke, & Ryan, 2011). If our findings are indicative of veridical memory recall, what neural processes might underpin such mnemonic stability? One candidate is reconsolidation (Nader, 2015), which refers to the restabilization of previously consolidated memory traces following reactivation, and it is possible the initial recall of the memories instantiated this process. In addition, consolidation can help improve memory for recent experience (Schapiro et al., 2017), particularly if those memory traces are weak (Schapiro, McDevitt, Rogers, Mednick, & Norman, 2018). It is therefore possible that offline neural reactivation of events (Tambini & Davachi, 2013) occurred after the first visit to stabilize representations further.

One potential consideration is whether the preserved memory details during Visit 2 reflect genuinely veridical memory recall. Recent theoretical consideration of the role of the hippocampus in remote memory retrieval raises the possibility that this may not be the case. Based on cross‐species evidence that hippocampal memory traces fade rapidly over time, Barry and Maguire (Barry & Maguire, 2019a, 2019b, but see Moscovitch & Nadel, 2019) have proposed that the hippocampus reconstructs remote memories by assembling relevant consolidated elements from the neocortex into coherent scenes. This reconstructive approach can account for the incorporation of plausible information into autobiographical memories (Pezdek, Blandon‐Gitlin, & Gabbay, 2006), particularly when they are remote (Barclay & Wellman, 1986). Some autobiographical memory details produced during the second visit may therefore reflect imagined aspects of experience. The inability to know with certainty whether memory details are a reflection of the ground truth is a limitation common to all studies investigating personal, real‐world autobiographical memories which have been subjectively experienced long before an experiment took place. However, given the highly specific nature of the internal details which were generated, and that our participants were not prompted with additional details, which is often observed in studies of false memory (Pezdek et al., 2006), on balance we believe it is more likely that the details provided during both visits reflected aspects of the original experience.

Closer inspection of the subcategories of details recalled revealed that in fact not all details were preserved over an 8‐month period. Subjective thoughts and emotions details were consistently vulnerable to decay over time. As depicted in Figure 3f, most participants produced fewer of these details, to varying degrees, at Visit 2 compared to 8 months previously. A representative “thought” detail was “I thought this was a great experience,” and an “emotion” detail was “I felt really happy.” Why might such details decline over time? Consolidation appears to selectively favor neutral over pleasant stimuli (Cellini, Torre, Stegagno, & Sarlo, 2016), and given that the recalled memories in this study were more positive than neutral in nature, this may explain why these particular aspects of experience were more likely to fade. The decay of subjective thoughts and emotions details was attenuated by a relatively larger pre/parasubiculum volume, which we consider next.

In line with one of our predictions, the neuroimaging findings showed a strong association between the amount of autobiographical memory details and pre/parasubiculum volume. This result may indicate that Palombo et al.'s (2018) previous report of an association between left subiculum volume (which encompassed the pre/parasubiculum in their protocol) and internal details was driven specifically by the pre/parasubiculum. It should be acknowledged that the inter‐rater agreement for delineating the pre/parasubiculum was lower than for most of the other subfields, and this highlights the challenge of studying this small area. However, the values are similar to those reported elsewhere in the literature (e.g., Dalton, McCormick, De Luca, et al., 2019; Dalton, McCormick, & Maguire, 2019).

Our potential pre/parasubiculum finding is consistent with an expanding body of research implicating this brain region in autobiographical memory recall and other functions. fMRI studies have revealed specific activation of the pre/parasubiculum during the retrieval of autobiographical memories (e.g., Addis et al., 2012). Its engagement has also been observed during the imagination of events, whether situated in the past or the future (Addis, Pan, Vu, Laiser, & Schacter, 2009).

As alluded to previously, a common process underlying imagination, past and future thinking may be the mental construction of scene imagery (Barry & Maguire, 2019a, 2019b; Hassabis & Maguire, 2007; Maguire & Mullally, 2013). In fact, compared to other hippocampal subregions, the pre/parasubiculum is most consistently active during scene construction, whether novel or recalled (Hassabis, Kumaran, & Maguire, 2007; Zeidman et al., 2015; Zeidman & Maguire, 2016). That it may be especially tuned to processing scenes was further emphasized by the study of Dalton et al. (2018). During fMRI, they had participants gradually build scene imagery from three successive auditorily‐presented object descriptions and an imagined 3D space. This was contrasted with constructing mental images of non‐scene arrays that were composed of three objects and an imagined 2D space. The scene and array stimuli were, therefore, highly matched in terms of content and the associative and constructive processes they evoked. The pre/parasubiculum was particularly engaged by the construction of scene imagery. Of note, Dalton et al. (2018) further found that 3D space alone (without objects) did not engage the hippocampus, including the pre/parasubiculum (see also Zeidman, Mullally, Schwarzkopf, & Maguire, 2012 for a similar result). Rather it seems to be the combination of objects/environmental features/landmarks with a 3D space that forms a scene and this is what consistently engages the pre/parasubiculum.

The pre/parasubiculum preferentially receives input from areas involved in visuospatial processing—the inferior parietal lobule, the posterior cingulate cortex and the retrosplenial cortex, which may explain its consistent role in the processing of scenes (Dalton & Maguire, 2017). Taking these observations into consideration, the current results suggest that individual differences in the amount of autobiographical memory details generated over time may relate to the capacity for constructing rich, spatially coherent scene imagery. In support of this idea, a recent individual differences study involving a large sample of participants (*n* = 217), found that the ability to construct scene imagery fully mediated the relationships between autobiographical memory recall and other hippocampal‐dependent functions such as future thinking and spatial navigation (Clark et al., 2019). Moreover, in the same sample, strategies involving scene imagery predominated when recollecting autobiographical memories (Clark, Monk, & Maguire, 2020). It is also notable that the volume of the pre/parasubiculum is of particular clinical importance as a diagnostic marker. This is because, unlike other hippocampal subfields, its size remains stable across the lifespan (Zheng et al., 2018), yet it is severely affected in Alzheimer's disease (Iglesias et al., 2016), and this atrophy is associated with memory recall ability (Lim et al., 2013).

The association between pre/parasubiculum volume and the persistence of memory over time was observed only in the left hemisphere. This echoes the effects of damage to, or removal of, hippocampal tissue. Left, as opposed to right, temporal lobectomy patients are impaired at recalling contextual aspects of episodes (Spiers et al., 2001), and autobiographical memory deficits scale with the extent of left hippocampal atrophy in patients with schizophrenia (Herold et al., 2013). In healthy people, left‐lateralized activation is more common in the medial temporal lobe and hippocampus during autobiographical memory retrieval as measured by fMRI (e.g., Addis, Wong, & Schacter, 2007; Hirshhorn, Grady, Rosenbaum, Winocur, & Moscovitch, 2012; Maguire, 2001; Miró et al., 2019; Svoboda, McKinnon, & Levine, 2006), and the simulation of future events (Campbell, Madore, Benoit, Thakral, & Schacter, 2017). Intracranial electrode recordings in the hippocampi of patients being evaluated for epilepsy surgery have confirmed this left‐sided dominance, with theta oscillations during encoding predicting subsequent episodic memory recall (Miller et al., 2018). Coherent activity between the left hippocampus and prefrontal cortex has also been revealed during magnetoencephalography (MEG) autobiographical memory retrieval (Fuentemilla, Barnes, Duzel, & Levine, 2014; McCormick, Barry, Jafarian, Barnes, & Maguire, 2020), and when people construct novel scene imagery (Barry, Barnes, Clark, & Maguire, 2019). Together these data suggest that autobiographical memory retrieval may rely more heavily on the left hippocampus.

Given the previous result of Palombo et al. (2018), we also hypothesized there would be a positive association between the volume of the left DG and/or CA3 and the ability to produce autobiographical memory details across an extended delay. While we observed a positive trend between the volume of the right CA3/2 region and mnemonic persistence, this did not pass the corrected statistical threshold. Nevertheless, there are reasons to suspect that CA3/2 may be involved in the preservation of event details over time. High resolution fMRI has revealed this region is engaged during “pattern completion,” the retrieval of a multi‐element event based on partial cue information (Grande et al., 2019). In the current study, participants were given a partial cue (the photograph), and asked to recall the entire event. Further evidence from patients with specific damage to CA3 show that the ability to produce details from autobiographical memories across the lifespan is impaired, indicating it is involved in the reconstruction of events which have taken place long ago (Miller et al., 2020). Therefore, the modest association observed here between the size of CA3/2 and the reproduction of memory details over a long time period may reflect individual differences in the ability to retrieve entire events based on limited information, although this should be interpreted with caution as the association did not survive statistical correction.

Our study also provided an insight into the nature of the memory details produced, and possibly preserved, across time and their links with hippocampal subregion volumes. Pre/parasubiculum volume was associated with the amount of details relating to specific events, perceptual features and, as noted previously, thoughts and emotions, with a comparable trend observed for place references. However, no such relationship was observed for time details. This was unexpected, as it has been suggested that the left hippocampus encodes temporal information about real‐world memories (Nielson, Smith, Sreekumar, Dennis, & Sederberg, 2015). It is possible that our study design may have reduced participants' reliance on temporal information. Temporal context was specified in advance by the selection of memories from eight time‐points. Furthermore, participants were asked to recall a specific temporally‐constrained event. Consequently, participants generated less than one temporal reference per memory.

As well as the pre/parasubiculum being challenging to delineate from MRI brain scans, another limitation of the current study was the modest sample size of 16 participants, and so the results should be regarded as preliminary until they are replicated in a larger sample. However, we would emphasize the value of having such detailed measures of autobiographical memories, sampled longitudinally over a lengthy time‐scale, combined with the detailed manual segmentation of hippocampal subregions. This gave an insight into ecologically valid, time‐dependent processes which are mostly absent in traditional laboratory‐based tests of episodic memory. The effects we report were strong and specific, but future studies are required to test their robustness. Further work is also needed to explore if individuals who produce more memory details over time are doing so with high accuracy. This could perhaps be achieved by using staged autobiographical memory events in the real world or in virtual reality, where the event details are known to the experimenter. In addition, in order to provide a more comprehensive picture of the neural processes underlying memory persistence, the connectivity of the pre/parasubiculum with other regions in the autobiographical memory retrieval network also needs to be examined in more detail, both structurally and functionally (see Dalton, McCormick, & Maguire, 2019, for a recent initial example).

To conclude, we have expanded on existing functional evidence by highlighting a link between left pre/parasubiculum volume and autobiographical memories. This appears to not only protect against memory decay, but may possibly enhance memory persistence, inviting further scrutiny of the role of this brain region in remote autobiographical memory retrieval.

## Data Availability

Requests for the data can be sent to e.maguire@ucl.ac.uk. All test materials and code used are published and open access.
